# Perceptual assessment of the quality of urban space - validation of criteria and metrics by older citizens

**DOI:** 10.3389/fpubh.2025.1676659

**Published:** 2025-12-19

**Authors:** Agnieszka Ptak-Wojciechowska, Agata Gawlak

**Affiliations:** Institute of Architecture and Heritage Protection, Faculty of Architecture, Poznan University of Technology, Poznań, Poland

**Keywords:** workshops, validation, perceptual assessment, quality of urban space, older citizens

## Abstract

Despite the urban challenges related to population aging and urbanization, cities remain insufficiently adapted to the needs of seniors. Numerous instruments and studies for the assessment of urban quality of life (QoL) have already been developed, but tools evaluating the age-friendliness of urban areas and involving participatory multi-criteria decision analysis (MCDA) are scarce. The study aimed to conduct workshops with the participation of seniors to validate the criteria, sub-criteria, and metrics used to assess the quality of senior-friendly architectural and urban space, formulated in previous studies on QoL and MCDA. The group task involved proposing metrics for assessing the quality of architectural and urban spaces by describing them on sticky notes, which were subsequently attached to specific sub-criteria on B1 boards. Participants were asked to select the three most important criteria and three most important sub-criteria on an A3 board. The answers were developed during group work, and were individually analyzed and compiled in a larger group. Both existing and newly proposed metrics were validated. The questionnaire survey, constituting a part of the assessment framework, was improved based on added/reformulated metrics. The most significant criterion, according to seniors, is ex aequo K1. *Accessibility of urban area for* aging *population* and K.6. *Adaptability for seniors* aging *in place*. Metrics repeatedly suggested were *lifts*, *handrails*, *benches*, as well as *proximity to shops and parks*. Urban planners and older citizens can use the questionnaire to evaluate the quality of urban spaces. The weights assigned by seniors suggest what aspects should be paid attention to when planning cities. Further research will include validation of the evaluation framework with experts and subsequently improving the questionnaire. The following stage will concern conducting a large-scale questionnaire survey among older residents.

## 1 Introduction

Even though cities around the world face challenges related to urbanization and the growing number of populations over the age of 65, urban spaces remain insufficiently adapted to the needs of older citizens (1–4). Since the urban environment has an impact on the quality of life (QoL) in the city, more and more institutions and researchers are developing appraisal instruments that allow us to analyze the space we live in (5–9). The results of such analyses, presented for instance, in the form of a ranking, gain media coverage and draw public and governmental attention (10, 11). Thus, it is worth developing urban tools that could enable drawing informative conclusions, allowing decision-makers to improve the urban structure. After all, instead of being a source of competition, rankings should serve as a good reference (12).

Although these cities differ significantly from one another, in many ways they are subject to very uniform evaluation criteria (9). Cities are also diverse within their own administrative boundaries, for instance, at the neighborhood level (13, 14). Yet still, comparisons in available rankings are mainly global, while they should also be local.

In the assessment of the urban QoL, more attention should be paid to subjective indicators regarding the perception of citizens, and a complete tool should examine both objective and subjective aspects (15, 16). Researchers studying residential satisfaction among seniors highlight the need to concentrate on the intricate interactions between individuals and environments (17, 18). According to the concept of Person-Environment Fit and Kahana’s theory, the perception of older adults of the living environment may be influenced by their personal preferences, concerning *physical amenities/esthetics, resource amenities, safety, stimulation/peacefulness, homogeneity/heterogeneity, and interaction/solitude*.

*Physical amenities/esthetics* refer to the positive physical characteristics of the environment, such as well-kept buildings or the surrounding greenery. *Resource amenities* include the availability of services in proximity. According to the authors, the need for esthetics is almost universal, while in the case of proximity, there may be individual differences in preferences. In turn, how the *safety* affects the perception of space can vary significantly depending on the individual and their life experiences. *Stimulation versus tranquility* takes into account the complexity of the built environment (movement, sounds, smells), as well as the degree to which this environment encourages self-expression or activity, responding to the physical requirements of individuals, their sensory and cognitive abilities. For example, for some seniors, a complex environment will be preferred, for others, it will cause stress. *Homogeneity/heterogeneity* refers to the diversity of the surrounding community. Some seniors will prefer to live among people of a similar age, while others, on the contrary, will want to integrate with various social groups. Finally, *interaction/solitude* is related to the level to which an environment encourages social interaction or, conversely, provides privacy. Some seniors need constant contact with people, while others value personal space highly (18).

To respond to the identified gaps, guidelines for a new instrument were developed in our previous studies, taking into account socio-cultural and demographic aspects, the urban tissue, and transparent multi-criteria decision analysis (MCDA) methodology, providing readable results that would be easy to interpret by decision-makers (19, 20).

The selection of assessment criteria is equally important as evaluation (21). Participatory MCDA fosters inclusiveness and transparency through engagement of the main groups interested in the solution in defining the criteria (22–24). While acknowledging the role of stakeholders in improving the implementation of decisions, studies concerning participatory MCDA in an age-friendly city are scarce. One of the most relevant studies is a study conducted by Raut (25), where the MCDA age-friendly criteria and sub-criteria were formulated taking into consideration the effects of a pilot study and focus group. The stakeholders were experts and respondents who were not older adults. Other studies involved engagement of the older adults in the process of defining assessment criteria. Ertz et al. conducted a workshop with the older adults to develop a set of factors influencing pedestrian route choices, namely passability, obstacles on the path, surface problems, security, sidewalk width, and slope (26). Curl et al. developed an audit checklist to assess outdoor fall risks based not only on literature but also on the experiences of people aged 65 years and above (27). Both studies are limited to specific urban problems and do not use MCDA. A closely related study is that of Moura et al., involving stakeholders and decision makers in the process of indicator selection and weighting (28, 29). However, seniors constitute only one of the four pedestrian groups, and the evaluation is narrowed down to walkability.

To the best of the authors’ knowledge, there is no study where both architects and seniors are involved as groups of decision-makers in the process of formulating criteria and metrics for a comprehensive city’s spatial structure assessment.

Thus, the following research questions were formulated:

*RQ1*: In what way could the proposed criteria, sub-criteria, and metrics be validated?

*RQ2*: What does the validated questionnaire for seniors, measuring their perception of a city’s spatial structure, look like?

*RQ3*: Which aspects of the spatial structure of the city are significant for seniors?

This study aims to validate the criteria, sub-criteria, and metrics for assessing the quality of architectural and urban space, and related to older people’s perspective, that were formulated in our previous study.

## 2 Materials and methods

The original tool was proposed based on the analysis of related works, such as 24 international and Polish available assessment instruments (e.g., rankings, guides, surveys, and created in the years 2007–2021), as well as 14 assessment models proposed by scientists (12, 13, 30–65). Eligibility of the selected tools was related to the scope of the research and the characteristics of the metrics included.

**Figure 1:**
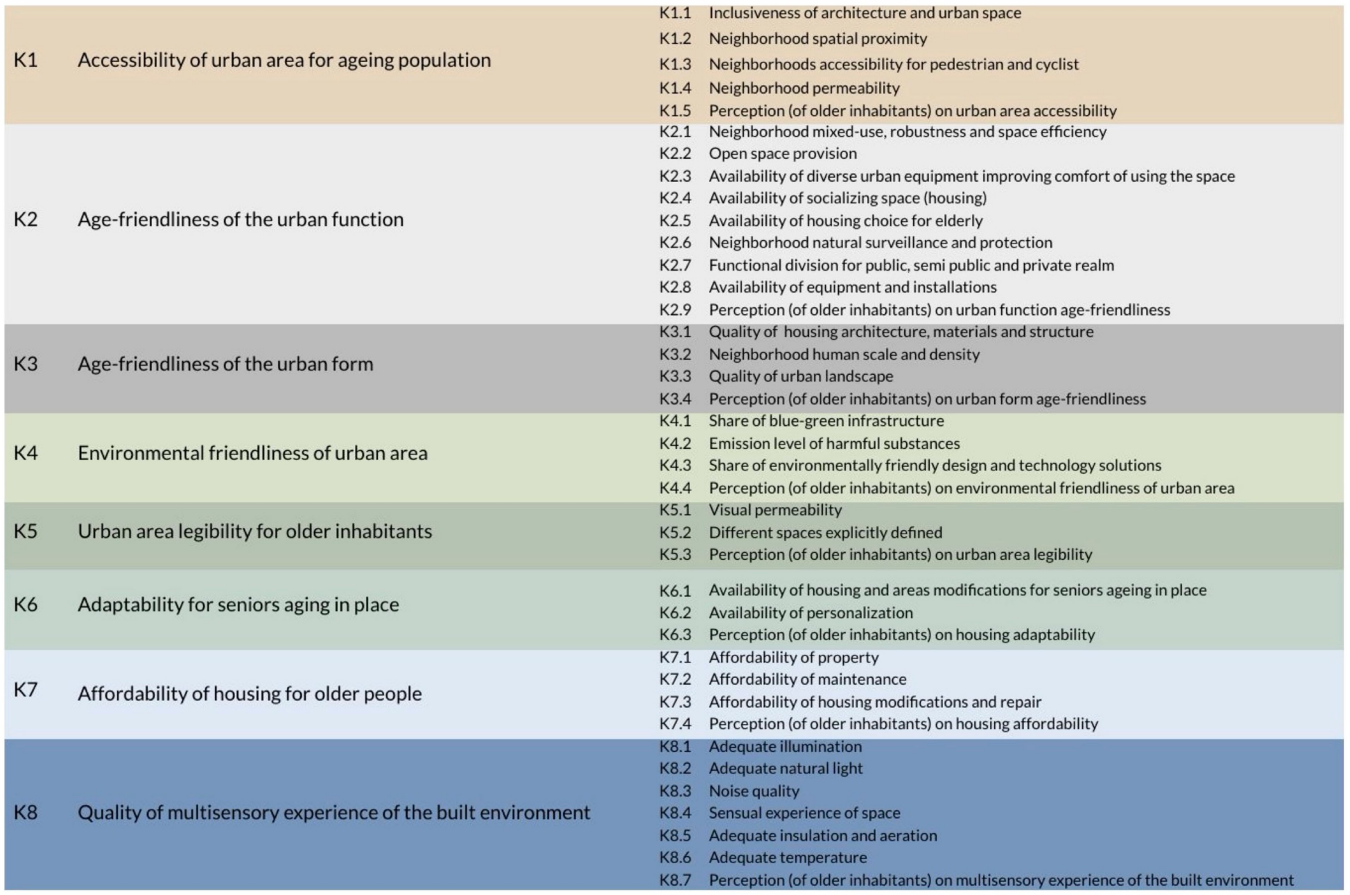
Final set of evaluation criteria and sub-criteria.

As a result of the analysis, 2,189 metrics, constituting a component of sub-criteria, and understood as the smallest element utilized to evaluate urban QoL, were obtained. Finally, 128 relevant metrics were included, after excluding metrics not related to the assessment of the spatial structure affecting the QoL and after removing redundancies. At the following stage, metrics were grouped within 39 sub-criteria, in accordance with the principle of “two pairs of eyes,” and analyzed literature. Differences in interpretation were discussed until a consensus was reached. Eventually, they were assigned to the main eight criteria, identified as the most effective for the study (Figure 1). The classification of metrics into criteria and sub-criteria was informed by two key frameworks: *WHO Global Age-friendly Cities. A guide* and *Responsive environments. A manual for designers* (43, 58), as well as Miller’s number (7 ± 2) concerning the cognitive limits of processing information.

To fill the gap, as available tools often do not combine the evaluation of different stakeholders, such as experts and seniors, each criterion of the original tool consisted of one sub-criterion aimed at the subjective evaluation of older residents, as well as sub-criteria meant for measurement by experts.

**Figure 2:**
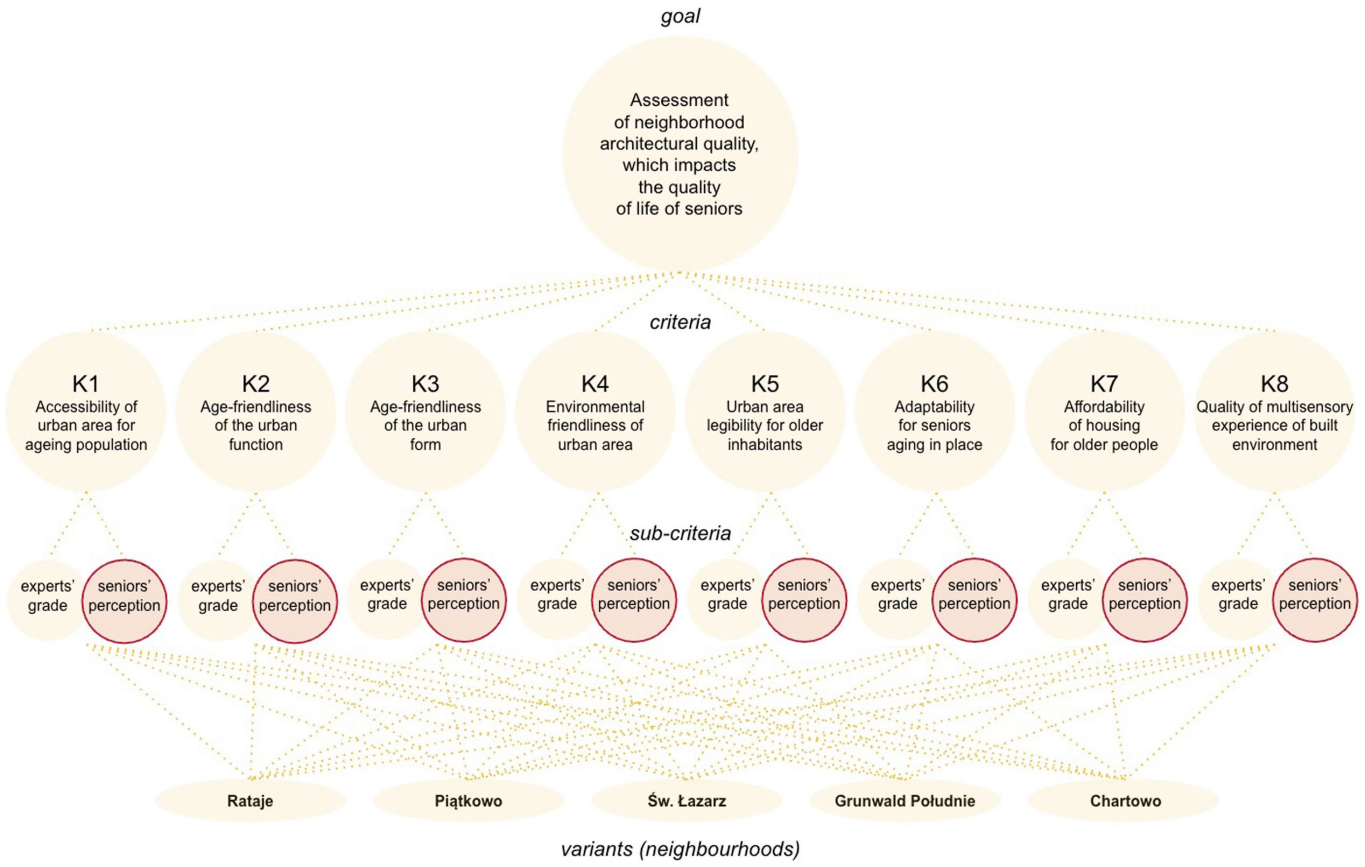
Decision tree with highlighted perceptual sub-criteria, developed by the authors using the AHP framework (68).

A transparent, user-friendly multi-criteria method, called the analytic hierarchy process (AHP), was selected (20, 66–68). It enabled the inclusion of differently measured sub-criteria (separate evaluation scales for experts and seniors) as shown in the decision tree (Figure 2). Its procedure consisted of the following steps: defining the decision problem and variants, selecting assessment criteria based on the analysis of 24 instruments and 14 assessment models, assigning weights to individual criteria and sub-criteria by experts through pair-wise comparison on 1–9 scale, evaluating variants against the criteria and their weights, calculating synthetic indicators for variants, generating the final ranking of variants. As decision variants, the Poznan neighborhoods inhabited by the largest number of seniors were selected.

Both experts and seniors received separate questionnaires for the assessment of neighborhoods (variants). Experts evaluated all five neighborhoods, while seniors evaluated only the neighborhood they are in. The questionnaire for seniors underwent a four-stage validation process, including expert consultations, a pilot study among seniors regarding its readability and evaluation by a psychologist, expert approval, and a positive opinion by the Committee for Ethics. It was conducted online via the senior initiative center and personally by visiting senior clubs.

In the questionnaire for seniors, three spatial scales were distinguished:

- *Housing* - understood as the interior of the inhabited building, including the balcony and garden.
- *Immediate surroundings* - understood as a 5-min walk from the apartment/house.
- *Further surrounding* - understood as the administrative boundaries of the neighborhood.

The effects of the discussed part of the study, which is described fully in (20, 66), were, among other things, a ranking of neighborhoods and recommendations for an appraisal instrument.

**Figure 3:**
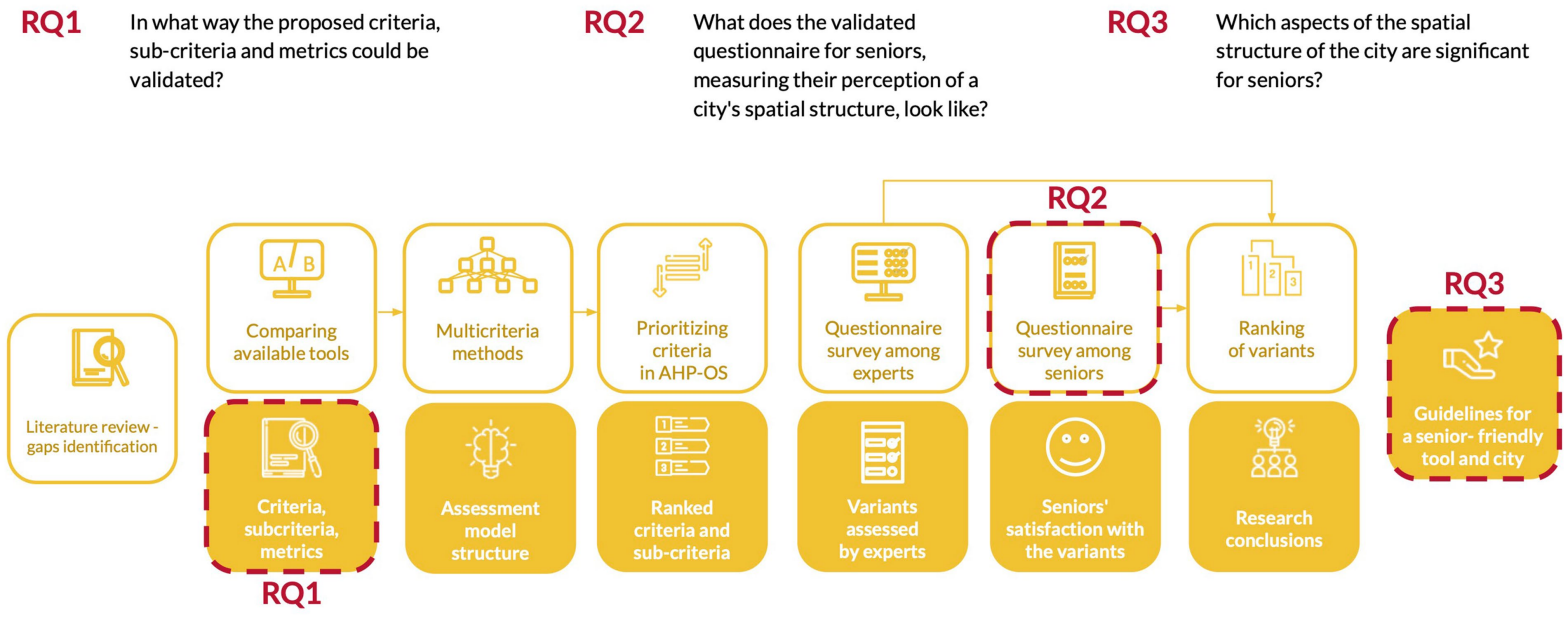
Overview of the research process with highlighted research questions.

The current study aimed to validate criteria, sub-criteria, and metrics used to assess the quality of senior-friendly architectural and urban space, formulated in previous studies, and to respond to the research questions (Figure 3). To achieve it, the study was organized as a workshop involving 25 seniors and assisted by five students from the Faculty of Architecture.

### 2.1 Sample

Older participants were recruited from a non-profit organization that conducts activities for various social groups, including older adults. Inclusion criteria were as follows: informed consent, age 65 or above, residing in the city, ability to participate independently, and without significant mobility limitations.

The study sample consisted of 23 women (92%) and 2 men (8%) with a mean age of 73.6 (Me = 73; SD = 4.01; range 68–85). The limited number of male participants is a limitation of the study.

### 2.2 Workshops

**Figure 4:**
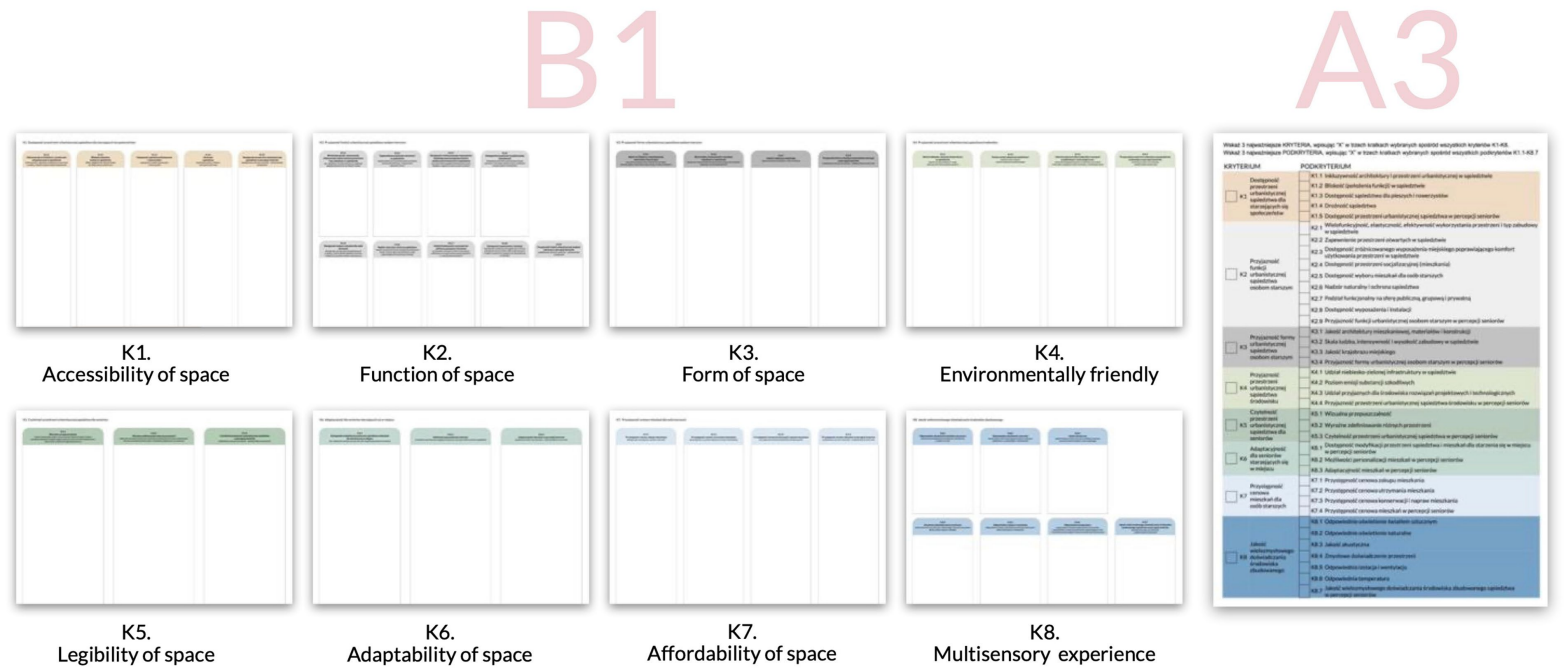
Materials used during workshop to collect data from seniors.

For each sub-criterion, we have prepared a separate B1 board (100×70 cm), while for prioritizing criteria and sub-criteria, another A3 board (Figure 4). Both the form of the study and the materials received a positive opinion from the Committee for Ethics.

The B1 board involved sub-criteria of the given criteria. The columns were marked in the colors assigned to each criterion. The smallest font used on the B1 board was size 20, while on the A3 board - 14.

The A3 board consisted of a tabular structure with four columns: the first column contained checkboxes for the main criteria, followed by the second column listing the names of these criteria. The third column included checkboxes corresponding to sub-criteria, and the fourth column listed the names of these sub-criteria (based on the set of criteria visible in Figure 1).

The workshop was preceded by a lecture introducing the topic and explaining the purpose of the study. Participants were informed that the results would be anonymized and analyzed within a group, and that participation was voluntary.

**Figure 5:**
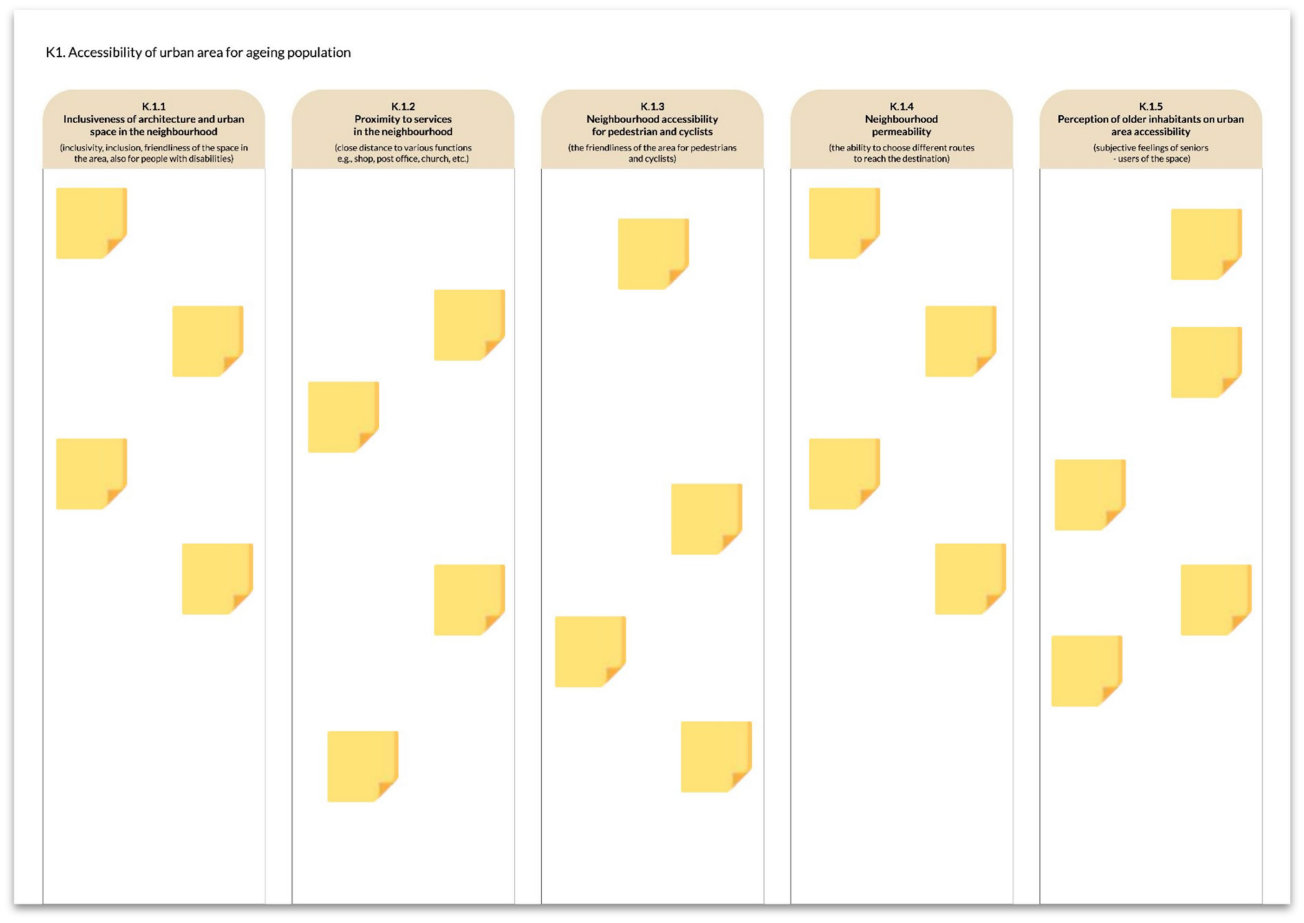
Exemplary B1 white board with sticky notes.

To address RQ1 and RQ3, the first part of the workshop covered proposing metrics aimed at assessing the quality of architectural and urban planning aspects. The seniors were divided into four tables, each suggesting metrics for all criteria and sub-criteria. Older adults described metrics on sticky notes and attached them to a 100×70 cm board within a specific evaluation sub-criterion (Figure 5). The second phase was the individual selection of the 3 most important criteria and sub-criteria for final prioritization by older participants. To make the study understandable for older participants, we have chosen a simple binary scoring system. Each selection was assigned a value of 1, while non-selected items received a value of 0. The scores were then summed for each criterion and sub-criterion and divided by the total number of responses, resulting in weights ranging from 0 to 1.

Since the suggested metrics were manually written on sticky notes attached to the B1 board, the first step in data processing was to capture them in the form of a photograph. The A3 boards were scanned. The data were then transcribed into an Excel spreadsheet. The metrics were initially assigned based on seniors’ opinions. Because we had defined the criteria and sub-criteria in previous studies based on existing assessment tools and literature, those metrics that, according to the original definitions, should have been included in a different sub-criterion were transferred accordingly. Next, the redundancy of the metrics was verified, and if they were identical, they were aggregated, recording the number of repetitions.

To answer RQ2, the last phase of our methods was the improvement of the evaluation framework and questionnaire for measuring seniors’ perceptions by researchers, based on added (new) or reformulated (initial) sub-criteria and metrics, as well as added new questions about personal preferences in the introduction.

## 3 Results

**Figure 6:**
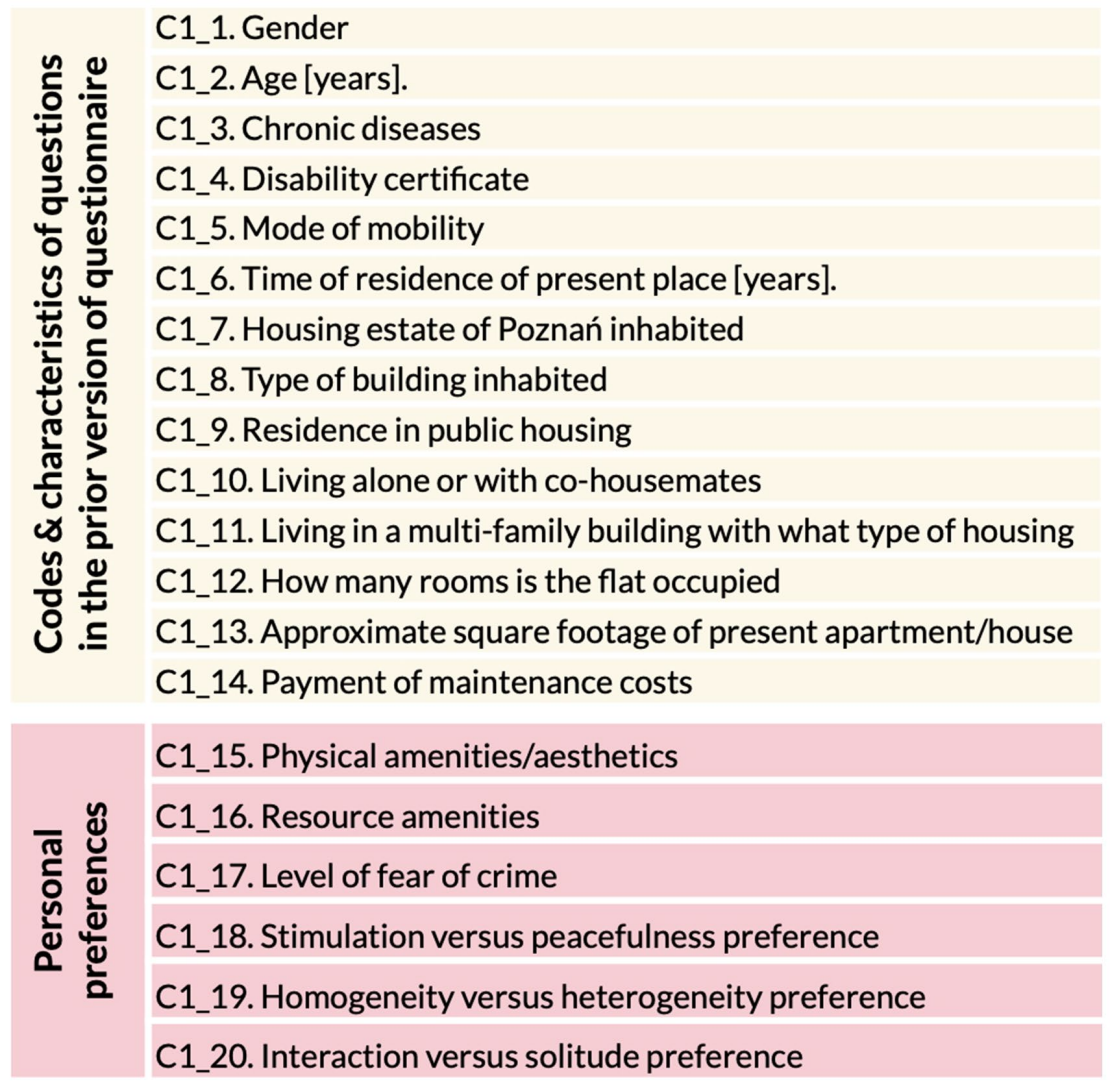
Introductory section of the questionnaire with added questions about personal preferences.

The results of our research are as follows. At first, based on literature studies, we have developed the introductory section of the questionnaire adding questions about personal preferences in terms of *Physical amenities/esthetics (C1_15), Resource amenities (C1_16), Level of fear of crime (C1_17), Stimulation versus peacefulness preference (C1_18), Homogeneity versus heterogeneity preference (C1_19),* and *Interaction versus solitude preference (C1_20)* (Figure 6).

Second, based on our workshops, we have formulated 285 metrics. Among the metrics, some relate to:

- Universal design principles: “*elevator in multi-story buildings,” “floors without carpets or rugs,” “bus stops adapted for disabled people,”* and *“Vienna-style tram stops* (on-street tram stop, where the road surface is elevated to the level of the sidewalk).*”*
- Assistive technologies: “*air conditioning,” “internet access,” “monitoring on the staircase,” “automatic switching on of lights,” “voice signals at pedestrian crossings,” “maintaining cleanliness,”* and *“installing devices that facilitate life activities.”*
- Other metrics are form-related: “*distance between buildings: blocks of flats are often so close that we can see into our neighbor’s window,”* and *“outdoor advertising.”*
- Function-related: “*need for medical assistance points in the building for seniors,” “lack of shelters for the population,” “outdoor gym,” “places to walk dogs,”* and *“accessibility of and proximity to swimming pool.”*
- Environmentally friendly related: “*urban water curtains* (a water-based system that uses pressurized water to spray a fine mist and cool the air).*”*

Metrics written down by participants were tallied. The frequency of each item suggests how often it is suggested. Metrics that were recurring at least three times were:

- lifts
- handrails/rails on walls in corridors and difficult-to-cross places
- proximity to shops
- lack of park/ proximity to park
- benches to sit along the way

Interestingly, some metrics were suggested in the form of what is needed (e.g., “*more lighting on streets and in parks”*), while others were in the form of a limitation (e.g., “*lack of building insulation”*). Not all metrics were assigned by seniors participating in a workshop according to our understanding of the given sub-criterion. Thus, we have revised them and organized them more systematically to ensure consistency with previously defined sub-criteria and enhance the logical structure.

Then, we obtained the importance of sub-criteria according to older adults (*n* = 15), with *K1.2. Neighborhood spatial proximity & K2.5. Availability of housing choice for the older adults* in the top place (27% *ex aequo*) and *K3.3. Quality of urban landscape & K7.1. Affordability of property* in the second place (20% *ex aequo*).

**Figure 7:**
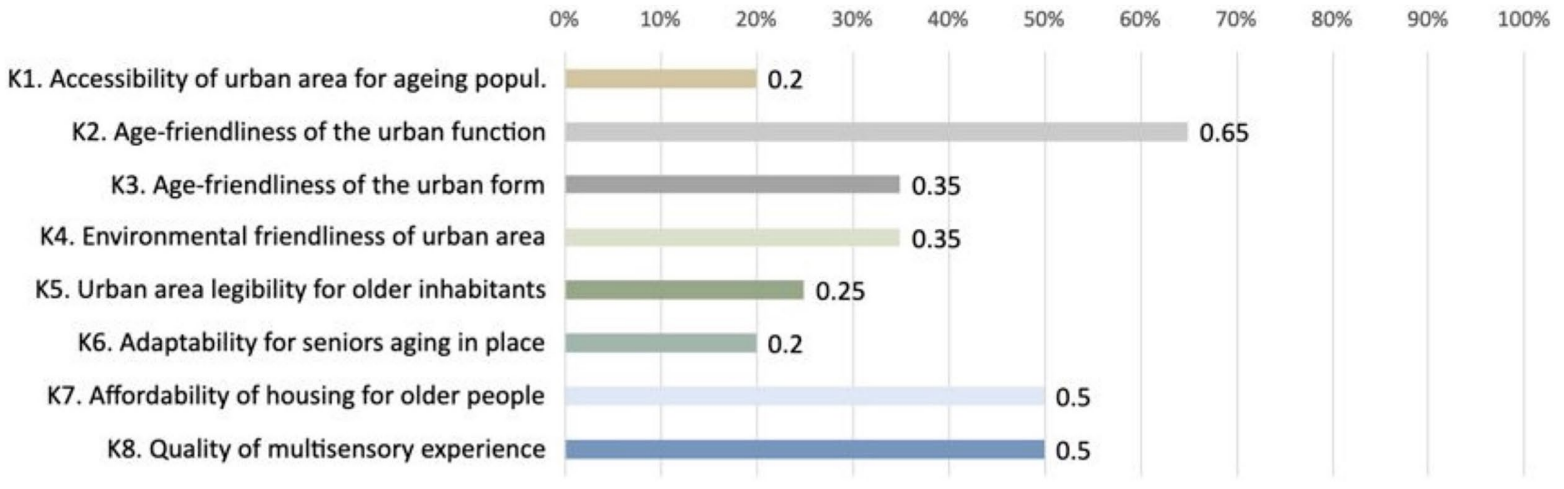
Horizontal bar chart presenting the importance of criteria according to seniors.

Another result was the importance of the main criteria according to older adults (*n* = 20): where the most important one turned out to be *K.2. Age-friendliness of the urban function* (65%), while the least important was *K.1. Accessibility of urban area for* aging *population (20%)* and *K.6. Adaptability for seniors* aging *in place (20%)* (Figure 7).

## 4 Discussion and conclusion

The study involved conducting workshops among older citizens, formulating new metrics, developing a validated questionnaire, and weighing spatial quality, including both criteria and sub-criteria.

In response to Research Question 1, we have presented a way to validate the part of the study concerning the perception of seniors, including a set of criteria, sub-criteria, and metrics. With regard to Research Question 2, we have extended the introductory part according to the concept of Person-Environment Fit, and we have developed a part including metrics (see Appendix). The study responded to Research Question 3, concerning aspects of the spatial structure of the city that are significant for seniors.

*Neighborhood spatial proximity,* ranked by seniors as the most important sub-criterion, together with repeatedly mentioned metrics, such as *proximity to shop* and *proximity to park,* are in line with our findings from the literature review, showing the significance of accessibility of urban services within 15-min walking time. On the other hand, the *availability of housing choices for the older adults*, ranked ex aequo, shows the need for a varied offer of residential units not only in size and layout, but also in view of different options of support. Among different types dedicated to an aging societies, we distinguish senior architecture, assisted living housing, controlled social mix, care farm, and synergic habitat (19, 69).

The fact that *benches* appeared frequently in participants’ responses corresponds to the results of earlier studies highlighting the importance of urban furniture, and specifically benches, for mobility and wellbeing of the older adults (70–73). Some metrics formulated during the workshops concern aspects of universal design and assistive technologies, which are in line with the gerontechnology and public health themes.

Some findings from this study should be viewed in light of its methodological limitations. For instance, a criterion *K1. Accessibility of urban areas for an aging population* was rated by seniors as least important, while in a previous study, the same criterion was evaluated as the most significant by experts (66). The possible reason may be adapting a binary scoring system and enabling older respondents only to choose from the three most important options. Another limitation may be a small sample size (*n* = 25) and a number of valid responses regarding prioritizing criteria (*n* = 20) and sub-criteria (*n* = 15). Finally, this workshop was conducted among older participants from a selected community, which may not encompass all relevant perspectives.

On the other hand, some unexpected results point to the importance of including perceptual quality in the assessment frameworks, since the objective expert assessment may differ significantly from the older people’s experience. According to Garcia et al., weighting can be assigned by a group of experts, which may result in more balanced values, but at the same time may not reflect the sensitivity of local users (13). Therefore, in their study, they use weighted importance resulting from experts’ opinions and older people’s subjective views. Although the study focuses on both the QoL of seniors and features of the urban environment, the number of indicators is limited to 2 main areas and 13 indicators. Seniors gave *Satisfaction with the accessibility of places* a rating of 3 out of 5. In the same study, *Satisfaction with basic needs services* (*supermarket, pharmacy, etc.*) was evaluated at 5.

A panel of 17 cross-sectional stakeholders, including, among others, senior citizens, scored the walkability key-concerns (28, 29). Two rounds of weighting with different selection methods (free range choice and constrained choice) were conducted. The weights were assigned differently, depending on the age group, the trip motive, and the selection method. For the leisure motive, seniors are rated highest with *C2: Convenience* (27%), consisting of *land use diversity, sidewalk effective width, daily commerce* (e.g.*, bakery*), *and services* (e.g.*, cash machine*). Conversely, *C1: connectivity*, consisting of *pedestrian infrastructure* (*path/sidewalk*) *continuity, path directness,* and *accessible pedestrian network,* was rated very low, ranking second from the bottom (7%). The difference in rating was smaller for the utilitarian motive, where C1 received 11% and C2 16%. Conducted in this study, pilot tests indicated that interviewees became distracted after approximately 30 min. This is in line with our approach, which aimed to simplify the weighting procedure.

Although MCDA and specifically AHP are used in studies related to topics of walkability, urban design qualities, and health (25, 36, 74), the evaluation criteria are most often formulated on the basis of literature, other tools, or expert opinions. Some studies involve co-creation, but they concern factors influencing pedestrian choices, spatial barriers, walkability, or novel interventions to support health, wellbeing, and independence (26–28, 75). The assessment most often concerns a selected urban problem, such as the quality of a street or a residential estate, and not a tool that combines various spatial scales (house, immediate vicinity, district), involves experts and seniors in co-creation and weighting, and is additionally based on a multi-criteria method.

**Figure 8:**
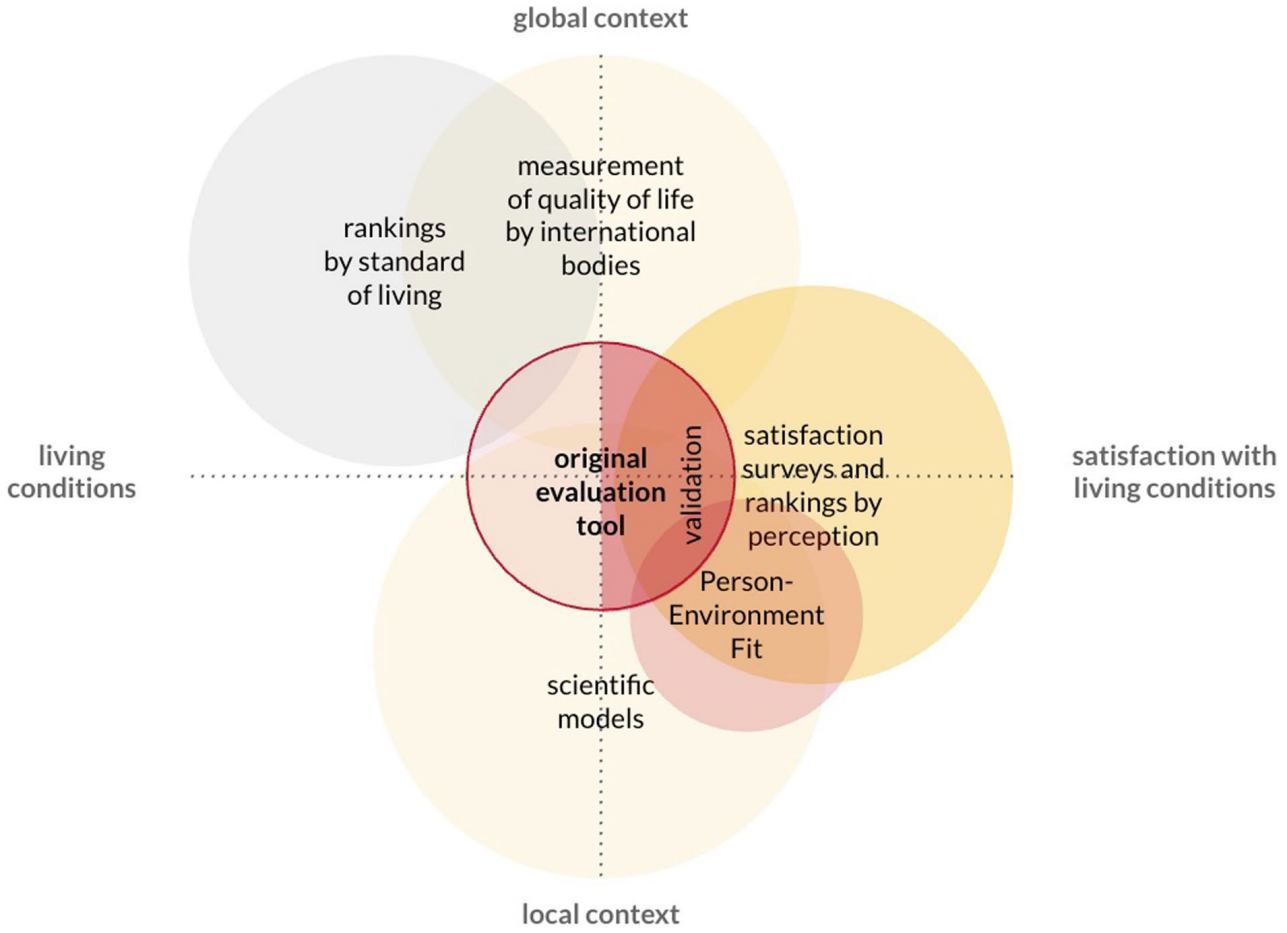
Continuation of research on the authorial evaluation tool – validation of the perceptual component.

The proposed method complements existing scientific tools and models (Figure 8). It allows taking into account both the global context and local conditions, and also includes the evaluation of the quality of space in the expert assessment and the level of satisfaction with it in the perception assessment of seniors. The new framework is more precise and pragmatic in terms of its application to creating a high urban quality of life. It may be used directly by seniors to evaluate how age-friendly their neighbourhood is. This method corresponds both to the scale of the city and its neighbourhoods.

The study provides valuable insights for urban planners and decision-makers who can use the questionnaire to evaluate the quality of urban spaces and their perceptual age-friendliness. The evaluation framework may be used as a checklist, while the weights assigned to criteria and sub-criteria may suggest which aspects of urban planning should be prioritized, according to seniors.

The avenue for future research is to validate the evaluation framework with experts, architects, and urban planners, to validate a newly developed section in the introduction of the questionnaire, including questions on personal preferences, and to conduct a large-scale questionnaire survey among older citizens. Responses of seniors, evaluating cities and neighborhoods/ districts in Poland, will be further analyzed with the use of not only statistics, but also machine learning methods.

## Data Availability

The raw data supporting the conclusions of this article will be made available by the authors, without undue reservation.
