# Supplementary material for: Perceptual assessment of the quality of urban space - validation of criteria and metrics by older citizens
Source: Front Public Health. 2025 Dec 19;13:1676659. doi: 10.3389/fpubh.2025.1676659 (PMC12758151; doi:10.3389/fpubh.2025.1676659)
Supplement: Supplementary file 1 [file Table_1.pdf]

Appendix

Tab. A.1. Seniors' questionnaire content.

Changes marked in color.

Questions	Answers
C1_1. Enter your gender	Man, Woman, Other
C1_2. Age	Years
C1_3. Do you suffer from chronic diseases?	Yes, No, I do not wish to specify
C1_4. Do you have a disability certificate? If so, to what extent?	I don't have disability,<br>Mild disability,<br>Moderate disability,<br>Severe/profound disability,<br>I do not wish to specify
C1_5. What is your mode of mobility?	Unaided,<br>In a wheelchair,<br>On crutches,<br>With a cane
C1_6. How long have you lived in your current place?	Years
C1_7. Which neighbourhood of Poznań do you live in?	Name of the neighbourhood
C1_8. Where do you live (type of building)?	Multi-family building, Single-family building, Retirement home
C1_9. Do you live in a in public housing?	Yes, No
C1_10. Do you live alone or with co-housemates?	Alone, With 1 (one) housemate,<br>With 2 (two) household members,<br>With 3 (three) household members,<br>With 4 (four) housemates,<br>With more household members (specify how many)
C1_11. If you live in a multi-family building, what type of housing is it about?	Block of flats, Tenement house, Block built after 2000, Modern multi-family building, Other type (specify which type)
C1_12. How many rooms is the flat you inhabit?	1 (one) room, 2 (two) rooms, 3 (three) rooms, 4 (four) rooms, Other (specify how many)
C1_13. Provide the approximate square footage of your current apartment/house	Less than 30 m2, 30 - 50 m2, 50 - 70 m2, 70 - 100 m2, 100 - 120 m2, Over 120 m2, I don't know
C1_14. Do you incur maintenance costs?	Yes, No
**C1_15. How important is the aesthetics of the living environment?**	**Not important;<br>Moderately important;<br>Very important**
**C1_16. How important is the proximity of resource amenities (shops and services) for everyday functioning?**	**Not important;<br>Moderately important;<br>Very important**
**C1_17. How important is the sense of security in the immediate environment?**	**Not important;<br>Slightly important;<br>Moderately important;<br>Very important;<br>Essential**
**C1_18. Which environment best suits your preferences?**	**Quiet and peaceful;<br>Moderately active (stimulating);<br>Active, vivid, providing many stimuli**
**C1_19. Which community in the environment suits you best?**	**Homogeneous (residents similar in terms of age, value, lifestyle);**

	**Diverse, heterogeneous (mix of people of different ages, values and lifestyles);**<br>**I don't pay attention to it**
**C1_20. What social conditions are the most comfortable?**	**Open relationships, ease of establishing contacts with other people;**<br>**Balance between contact and privacy;**<br>**Ability to maintain privacy and peace of mind**
C2_1. To what extent are you satisfied with the cost of maintaining the apartment/house you occupy (rent, utilities, loan amount)?	1, 2, 3, 4, 5
C2_2. To what extent are you satisfied with the availability of corridor and hallway in the apartment/house (e.g., door and corridor widths, non-slippery floor surfaces, **no carpets**)?	1, 2, 3, 4, 5
C2_3. To what extent are you satisfied with the adaptation of the apartment/house to the needs of people with disabilities (e.g., **is there an elevator if more than one story,** are there handrails in the bathroom)?	1, 2, 3, 4, 5
C2_4. To what extent are you satisfied with the furniture in your apartment/house (height of its installation, free use, unlimited access)?	1, 2, 3, 4, 5
C2_5. To what extent are you satisfied with the functionality of the apartment/house (do you have a place to sleep, rest, work, hygiene, cook)?	1, 2, 3, 4, 5
C2_6. To what extent are you satisfied with the level of furnishing of the apartment/house with the necessary furniture (e.g., bed, wardrobe, table, etc.)?	1, 2, 3, 4, 5
C2_7. To what extent are you satisfied with the equipment of your apartment/house (electronics - radio and TV, **Internet,** household appliances)?	1, 2, 3, 4, 5
C2_8. To what extent are you satisfied with the feeling of security in the apartment/house (do you feel safe, **is the building monitored: CCTV or security staff**)?	1, 2, 3, 4, 5
C2_9. To what extent are you satisfied with the aesthetics of the apartment/house (how much do you like it)?	1, 2, 3, 4, 5
C2_10. To what extent are you satisfied with the quality of the finishing materials (flooring, walls and ceilings)?	1, 2, 3, 4, 5
C2_11. To what extent are you satisfied with the technical condition of the apartment/house (e.g., solid structural elements, leak-proof floor and roof, rot-free window and door joinery)?	1, 2, 3, 4, 5
C2_12. To what extent are you satisfied with the use of environmentally friendly technologies in your apartment/house (e.g., solar panels, low-energy appliances, energy-saving light bulbs, etc.)?	1, 2, 3, 4, 5
C2_13. To what extent are you satisfied with the technical possibility of introducing amenities in the apartment/house (e.g., installing handrails, widening the passage, door)?	1, 2, 3, 4, 5
C2_14. To what extent are you satisfied with the hot water installation in your apartment/house (do you have hot water in the tap)?	1, 2, 3, 4, 5
C2_15. To what extent are you satisfied with the possibility of personalizing the apartment/house (the possibility of changing the color of the wall, accessories, paintings, curtains, etc.)?	1, 2, 3, 4, 5
C2_16. To what extent are you satisfied with the natural lighting of your apartment/house (sunlight)?	1, 2, 3, 4, 5
C2_17. To what extent are you satisfied with the illumination of your apartment/house with artificial light (lamps)?	1, 2, 3, 4, 5
C2_18. To what extent are you satisfied with the acoustics in your house/apartment (are you not bothered by the noise from the street and the neighbors)?	1, 2, 3, 4, 5
C2_19. To what extent are you satisfied with the temperature in your house/apartment (including the possibility of temperature control, is there air-conditioner)?	1, 2, 3, 4, 5
C2_20. To what extent are you satisfied with the possibility of ventilating your house/apartment?	1, 2, 3, 4, 5
C2_21. To what extent are you satisfied with the possibility of feeling the space through touch, sounds, smells?	1, 2, 3, 4, 5
**C2_22. To what extent are you satisfied with the technology that supports your daily life (e.g., fall detection technology, facilitation of communication with relatives/doctor or entertainment, automatically switching on stairwell lights, and more)?**	**1, 2, 3, 4, 5**
**C2_23. To what extent are you satisfied with the level of privacy in the apartment/house, resulting from e.g., the distance between buildings (no direct view of the neighbors' windows)?**	**1, 2, 3, 4, 5**
C3_1. To what extent are you satisfied with the possibility of free movement in the immediate vicinity 400 m from the dwelling/home (no obstacles, ramps, handrails at the external stairs)?	1, 2, 3, 4, 5

C3_2. To what extent are you satisfied with the availability of public buildings (e.g., offices, churches, stations, clinics, shops) in the nearest neighbourhood 400 m from the dwelling/home (sufficiently wide door openings and corridors, stairs at the entrance to buildings)?	1, 2, 3, 4, 5
C3_3. To what extent are you satisfied with the distance from your place of residence to service and commercial establishments, in the nearest neighbourhood 400 m from your dwelling/home?	1, 2, 3, 4, 5
C3_4. To what extent are you satisfied with the pedestrian routes and cycle paths in the in the nearest neighbourhood 400 m from the dwelling/home (non-slippery surfaces and no high curbs)?	1, 2, 3, 4, 5
C3_5. To what extent are you satisfied with the pedestrian routes and bicycle paths in the nearest neighbourhood 400 m from the dwelling/home (width of the pavements allowing for wheelchair passage or passage with crutches)?	1, 2, 3, 4, 5
C3_6. To what extent are you satisfied with the availability of pedestrian routes and bicycle paths in the nearest neighbourhood 400 m from the dwelling/home (safe pedestrian crossings, separation of pedestrian traffic from car and bicycle traffic)?	1, 2, 3, 4, 5
C3_7. To what extent are you satisfied with the possibility of choosing different routes (changing the route from the accommodation to the same place, e.g., a shop or a church) in the nearest neighbourhood 400 m from the dwelling/home?	1, 2, 3, 4, 5
C3_8. To what extent are you satisfied with the variety of functions in the nearest neighbourhood 400 m from the dwelling/home (shops, clinics, pharmacies, church, office, etc.)?	1, 2, 3, 4, 5
C3_9. To what extent are you satisfied with public spaces such as squares, markets, pedestrian streets in the nearest neighbourhood 400 m from your dwelling/home?	1, 2, 3, 4, 5
C3_10. To what extent are you satisfied with the number of benches in the nearest neighbourhood 400 m from the dwelling/home?	1, 2, 3, 4, 5
C3_11. To what extent are you satisfied with the form of the benches in the nearest neighbourhood 400 m from the dwelling/home (are the benches comfortable)?	1, 2, 3, 4, 5
C3_12. To what extent are you satisfied with the placement of garbage cans in the nearest neighbourhood 400 m from the dwelling/home?	1, 2, 3, 4, 5
C3_13. To what extent are you satisfied with the arrangement of bicycle parking in the nearest neighbourhood 400 m from the dwelling/home?	1, 2, 3, 4, 5
C3_14. To what extent are you satisfied with the feeling of security in the nearest neighbourhood 400 m from the dwelling/home (do you feel safe while walking down the street or in the park)?	1, 2, 3, 4, 5
C3_15. To what extent are you satisfied with the city's offer for seniors (**e.g., events, medical aid points or shelters, outdoor gyms, swimming pools, dog walking areas, etc.** ~~programs, concerts, festivals, other activities~~) in the nearest neighbourhood 400 m from the dwelling/home?	1, 2, 3, 4, 5
C3_16. To what extent are you satisfied with the aesthetics of the buildings in the nearest neighbourhood 400 m from the dwelling/home (do you like the facades of the buildings)?	1, 2, 3, 4, 5
C3_17. To what extent are you satisfied with the aesthetics of the nearest neighbourhood 400 m from the dwelling/home (e.g., clean and well-maintained streets and street greenery, **lack of advertising clutter**)?	1, 2, 3, 4, 5
C3_18. To what extent are you satisfied with the condition of the buildings in the i nearest neighbourhood 400 m from the dwelling/home (e.g., their maintenance, cleanliness of the facades)?	1, 2, 3, 4, 5
C3_19. To what extent are you satisfied with the green areas in the nearest neighbourhood 400 m from the dwelling/home (availability of parks, squares, and other greenery, e.g., green roofs, trees and other natural landscape elements)?	1, 2, 3, 4, 5
C3_20. To what extent are you satisfied with the water features in the nearest neighbourhood 400 m from the dwelling/home (river, lake, city fountains, **water curtains**)?	1, 2, 3, 4, 5
C3_21. To what extent are you satisfied with the air quality (during the heating season) in the nearest neighbourhood 400 m from the dwelling/home?	1, 2, 3, 4, 5
C3_22. To what extent are you satisfied with the readability of the space in the nearest neighbourhood 400 m from the dwelling/home (do you easily orient yourself in the space and find the right way)?	1, 2, 3, 4, 5
C3_23. To what extent are you satisfied with the daylight provided to public spaces, including streets, in the nearest neighbourhood 400 m from the dwelling/home?	1, 2, 3, 4, 5
C3_24. To what extent are you satisfied with the appropriate lighting of streets, pedestrian crossings, squares, facades of buildings with artificial light (lamps) in the nearest neighbourhood 400 m from the dwelling/home?	1, 2, 3, 4, 5
C3_25. To what extent are you satisfied with the shading elements (canopies, shelters, trees) in the nearest neighbourhood 400 m from the dwelling/home?	1, 2, 3, 4, 5
C3_26. To what extent are you satisfied with the tactile sensations in the nearest neighbourhood 400 m from the dwelling/home (different materials, textures, e.g., on the pavement, on the stairs, on station platforms)?	1, 2, 3, 4, 5

C3_27. To what extent are you satisfied with the noise level in the nearest neighbourhood 400 m from the dwelling/home?	1, 2, 3, 4, 5
C3_28. To what extent are you satisfied with the variety of forms, materials and textures used on buildings and streets in the nearest neighbourhood 400 m from the dwelling/home (e.g., paving stones, plaster, façade bricks, wood, concrete, etc.)?	1, 2, 3, 4, 5
**C3_29. To what extent are you satisfied with the accessibility of tram or bus stop space in the nearest neighbourhood 400 m from the dwelling/home?**	**1, 2, 3, 4, 5**
C4_1. To what extent are you satisfied with the ability to move freely in the further surroundings/neighbourhood (no obstacles, ramps, handrails at the external stairs)?	1, 2, 3, 4, 5
C4_2. To what extent are you satisfied with the accessibility of public buildings (e.g., offices, churches, stations, clinics, shops) in the further surroundings/ neighbourhood (sufficiently wide door openings and corridors, stairs at the entrance to buildings)?	1, 2, 3, 4, 5
C4_3. To what extent are you satisfied with the distance from your place of residence to service and commercial establishments, in a further environment/ neighbourhood?	1, 2, 3, 4, 5
C4_4. To what extent are you satisfied with the pedestrian routes and cycle paths in the further surroundings/neighbourhood (non-slippery surfaces and no high curbs)?	1, 2, 3, 4, 5
C4_5. To what extent are you satisfied with the pedestrian routes and cycle paths in the further surroundings/neighbourhood (width of the pavements allowing for wheelchair passage or passage with crutches)?	1, 2, 3, 4, 5
C4_6. To what extent are you satisfied with the availability of pedestrian routes and bicycle paths in the further surroundings/neighbourhood (safe pedestrian crossings, separation of pedestrian and car and bicycle traffic)?	1, 2, 3, 4, 5
C4_7. To what extent are you satisfied with the possibility of choosing different accesses (changing the route from your place of residence to the same place, e.g., a shop or a church) in the further surroundings/neighbourhood?	1, 2, 3, 4, 5
C4_8. To what extent are you satisfied with the variety of functions in the further surroundings/neighbourhood (shops, clinics, pharmacies, church, office, etc.)?	1, 2, 3, 4, 5
C4_9. To what extent are you satisfied with public spaces such as squares, markets, pedestrian streets in the further surroundings /neighbourhood?	1, 2, 3, 4, 5
C4_10. To what extent are you satisfied with the number of benches in the further surroundings/neighbourhood?	1, 2, 3, 4, 5
C4_11. To what extent are you satisfied with the form of the benches in the further surroundings/neighbourhood (are the benches comfortable)?	1, 2, 3, 4, 5
C4_12. To what extent are you satisfied with the location of garbage cans in the further surroundings/neighbourhood?	1, 2, 3, 4, 5
C4_13. To what extent are you satisfied with the arrangement of bicycle parking in the further surroundings/neighbourhood?	1, 2, 3, 4, 5
C4_14. To what extent are you satisfied with the feeling of security in the further surroundings/neighbourhood (do you feel safe while walking down the street or in the park)?	1, 2, 3, 4, 5
C4_15. To what extent are you satisfied with the city's offer for seniors (**e.g., events, medical aid points or shelters, outdoor gyms, swimming pools, dog walking areas, etc.** ~~programs, concerts, festivals, other activities~~) in the further surroundings/neighbourhood?	1, 2, 3, 4, 5
C4_16. To what extent are you satisfied with the aesthetics of the buildings in the further surroundings/neighbourhood (do you like the facades of the buildings)?	1, 2, 3, 4, 5
C4_17. To what extent are you satisfied with the aesthetics of the further surroundings/ neighbourhood (e.g., clean and well-maintained streets and street greenery, **lack of advertising clutter**)?	1, 2, 3, 4, 5
C4_18. To what extent are you satisfied with the condition of the buildings in the further surroundings/neighbourhood (e.g., their state of maintenance, cleanliness of the facades)?	1, 2, 3, 4, 5
C4_19. To what extent are you satisfied with the green areas in the further surroundings/ neighbourhood (accessibility of parks, squares, and other greenery, e.g., green roofs, trees and other natural landscape elements)?	1, 2, 3, 4, 5
C4_20. To what extent are you satisfied with the water features in the further surroundings/ neighbourhood (river, lake, city fountains, **water curtains**)?	1, 2, 3, 4, 5
C4_21. To what extent are you satisfied with the air quality (during the heating season) in the further surroundings/neighbourhood?	1, 2, 3, 4, 5
C4_22. To what extent are you satisfied with the readability of the space in the further surroundings/neighbourhood (do you easily orient yourself in the space and find the right way)?	1, 2, 3, 4, 5
C4_23. To what extent are you satisfied with the lighting of public spaces, including streets, with daylight, in the further surroundings /neighbourhood?	1, 2, 3, 4, 5
C4_24. To what extent are you satisfied with the appropriate lighting of streets, pedestrian crossings, squares, building facades with artificial light (lamps) in the further surroundings/ neighbourhood?	1, 2, 3, 4, 5

C4_25. To what extent are you satisfied with the shading elements (canopies, shelters, trees) in the further surroundings/neighbourhood?	1, 2, 3, 4, 5
C4_26. To what extent are you satisfied with the tactile sensations in the further surroundings/neighbourhood (different materials, textures, e.g., on the pavement, on the stairs, on the station platforms)?	1, 2, 3, 4, 5
C4_27. To what extent are you satisfied with the noise level in the further surroundings/ neighbourhood?	1, 2, 3, 4, 5
C4_28. To what extent are you satisfied with the variety of forms, materials and textures used on buildings and streets in the further surroundings/ neighbourhood (e.g., paving stones, plaster, façade bricks, wood, concrete, etc.)?	1, 2, 3, 4, 5
**C4_29. To what extent are you satisfied with the accessibility of tram or bus stop space in the further surroundings/ neighbourhood?**	
C5_1. To what extent do you identify with your immediate surroundings 400 m from your dwelling/home?	1, 2, 3, 4, 5
C5_2. To what extent do you identify with your surroundings within the boundaries of the neighbourhood you live in?	1, 2, 3, 4, 5
